# A Challenging Diagnosis of Sheehan’s Syndrome in Non-obstetric Critical Care and Emergency Settings: A Case Series of Five Patients with Varied Presentations

**DOI:** 10.2478/jccm-2022-0018

**Published:** 2022-08-12

**Authors:** Suhail Sarwar Siddiqui, Nibu Dominic, Sukriti Kumar, Kauser Usman, Sai Saran, Avinash Agrawal, Mohan Gurjar, Syed Nabeel Muzaffar

**Affiliations:** 1 Department of Critical Care Medicine, King George Medical University, Lucknow, Uttar Pradesh, India; 2 Department of Endocrinology, Aster Medcity, Kochi, Kerala, India; 3 Department of Radiodiagnosis, King George Medical University, Lucknow, Uttar Pradesh, India; 4 Endocrinology Division, King George Medical University, Lucknow, Uttar Pradesh, India; 5 Department of Critical Care Medicine, Sanjay Gandhi Post Graduate Institute of Medical Sciences, Lucknow, Uttar Pradesh, India

**Keywords:** Sheehan’s syndrome, hypopituitarism, pituitary necrosis, postpartum haemorrhage

## Abstract

Sheehan’s syndrome is a life-threatening endocrine emergency seen in postpartum females secondary to ischemic pituitary necrosis. It is a frequent cause of hypopituitarism in developing countries that occurs secondary to postpartum haemorrhage (PPH). Patients with Sheehan’s syndrome often present with organ dysfunctions in critical care settings, secondary to stressors precipitating the underlying hormonal deficiencies. The initial clinical picture of Sheehan’s syndrome may mimic some other disease, leading to misdiagnosis and diagnostic delay. Strict vigilance, timely diagnosis, and appropriate management are essential to avoid diagnostic delay and to improve the patient outcome. In this case series, we describe 5 cases of previously undiagnosed Sheehan’s syndrome (including young, middle aged and postmenopausal females) that presented to critical care and emergency settings with organ failures.

## Introduction

Sheehan’s syndrome is a life-threatening endocrine emergency resulting from ischemic pituitary necrosis in postpartum females [1,2]. It is an important cause of hypopituitarism in developing countries. Sheehan’s syndrome is seen in 1-2% of females, who lose up to 1-2 litres (L) of blood during postpartum haemorrhage (PPH), with subsequent hypotension [3,4]. In patients with hypopituitarism, the prevalence of Sheehan’s syndrome is around 6-8% [5]. Severity of disease depends upon the number of hormonal axes affected by destruction of pituitary gland. Sheehan’s syndrome generally occurs if 70-90% of the gland has been destroyed [6]. In many cases, initial symptoms are chronic and nonspecific, which often delays the diagnosis of Sheehan’s syndrome. A meticulous history and examination are thus essential to pick up these cases. Patients often end up with organ dysfunctions secondary to hormonal deficiencies, necessitating Intensive Care Unit (ICU) admissions. Timely diagnosis and management are pivotal to improve the outcome of patients [7]. We hereby describe 5 cases of previously undiagnosed Sheehan’s syndrome that presented to critical care and emergency settings with organ dysfunctions. Informed consent was taken from the patients for this case series, including the publication of MRI brain images, provided identity is not revealed.

## Case 1: Middle-aged postpartum female with seizures and altered sensorium

**Fig. 1:**
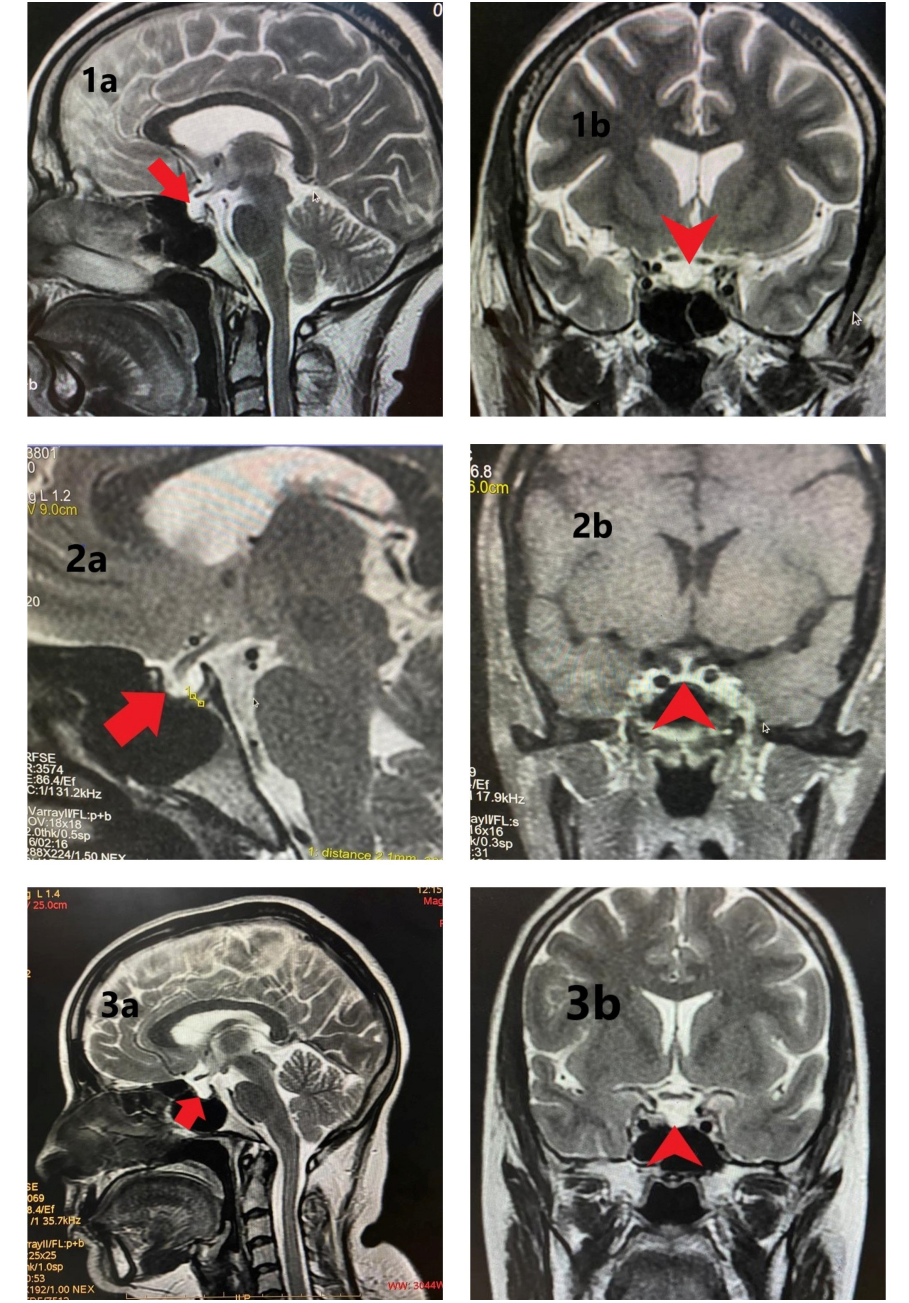
(a) Sagittal (red bold arrow) and (b) Coronal T2 weighted (red bold arrow-head) images showing sella filled with CSF and thinned and flattened pituitary (height reduced) lying against floor of sella - partially empty sella. Figure 2: (a) Sagittal T2 weighted (red bold arrow) and (b) Coronal post contrast T1 weighted (red bold arrow-head) images showing reduced pituitary height (2.1mm -as measured between calipers in 2a). Figure 3: (a) Sagittal (red bold arrow) and (b) Coronal T2 weighted (red bold arrow-head) images showing sella filled with CSF and pituitary gland severely thinned, lying flattened against sellar floor.

A 41-year-female got admitted to ICU with loss of appetite for 20 days, pain abdomen for 7 days and altered sensorium for 5 days. She had been treated with anti-tuberculous drugs ATT for the last 3 weeks due to a suspicion of tuberculous meningitis (TBM). On examination, she was disoriented, hemodynamically unstable (BP: 80/40mm Hg), bradycardic (heart rate: 50-60/min) but otherwise spontaneously breathing without oxygen supply. There were no focal neurological deficits. CSF workup was normal. Labs at admission revealed hyponatremia which was managed with 3% hypertonic saline infusion. 12 lead electrocardiography (ECG) showed sinus bradycardia and 2D echocardiography (ECHO) was suggestive of left ventricular (LV) dysfunction with ejection fraction (EF) about 40%. Intravenous (IV) fluid resuscitation, vasopressor support and empirical antimicrobials were immediately initiated, but no significant hemodynamic improvement had been reported. She had a past history of lactation failure, amenorrhoea and slowness of movements and speech following hysterectomy performed 12 years ago for PPH. Thus, in view of possible endocrine dysfunction, IV hydrocortisone was subsequently added, which alleviated the shock profile. Hormonal tests revealed secondary hypothyroidism, secondary hypocortisolism and decreased serum prolactin. Hyponatremia at admission could be secondary to underlying hypocortisolism and hypothyroidism. The ATT was interrupted because of no apparent evidence of Tuberculosis and as a prevention of liver dysfunction. Magnetic resonance imaging (MRI) brain showed CSF-like intensity in sella turcica with thinning and flattening of pituitary gland against floor of sella (Figure 1).

Sheehan’s syndrome diagnosis was established on the basis of the above clinical presentation and laboratory workups. The patient condition improved after steroid and oral levothyroxine substitution, and was subsequently discharged with oral prednisolone and levothyroxine prescription after 3 weeks of ICU stay.

## Case 2: Young postoperative female with left ventricular dysfunction

A 34-year-female (with a PPH history that required hysterectomy 3 years ago) was admitted to ICU in intubated condition after emergency laparotomy for pyoperitoneum. She had a history of PPH around 3 years ago, requiring hysterectomy for further management. In immediate postoperative period, there was prolonged hypotension and hypoglycaemia, for which she was shifted to Intensive Care unit (ICU). At ICU admission, management was initiated with broad-spectrum antimicrobials, fluid resuscitation and vasoactive drugs. Ultrasonography (USG) abdomen did not reveal any abdominal collection. Surgical wound was healthy. 2D echocardiography revealed LV systolic dysfunction. Due to ongoing hemodynamic instability and hypoglycaemia requiring intravenous dextrose infusion (with normal liver and kidney function tests), pituitary dysfunction was suspected and blood was collected for serum cortisol and pituitary hormonal profile testing; empirical IV hydrocortisone was added. Hypotension and hypoglycaemia improved after addition of steroids. Suspicion of pituitary dysfunction was thus kept and serum pituitary hormonal profile evaluated. Serum hormonal workup was suggestive of anterior pituitary hormone deficiency (secondary hypothyroidism, secondary hypocortisolism, low serum prolactin and low gonadotropins). Shock and hypoglycaemia were thus probably due to hypocortisolism. MRI brain (Figure 2) showed reduced pituitary height of around 2.1mm. Therapy was started with levothyroxine 50 mcg per 24 hours and infusion of hydrocortisone continued; that was later shifted to oral prednisolone.

The diagnosis of Sheehan’s syndrome was supported by the neurological and endocrine examination, and confirmed by the laboratory and radiological findings. The patient gradually improved and she was weaned off from ventilator and discharged.

## Case 3: Young postpartum female with anxiety, headache and anorexia

A 28-year-female presented to emergency room (ER) in altered sensorium, bradycardia and hypotension. She had a past history of PPH after full term normal vaginal delivery around 2 years ago, after which she developed amenorrhoea and lactation failure. In addition, she complained of frequent episodes of anxiety, headache, giddiness, anorexia, generalized body aches and restlessness. At admission, she was pale, drowsy, bradycardic (pulse rate: 60/min), hypotensive (BP: 80/60), but spontaneously breathing without oxygen supply. ECG was suggestive of sinus bradycardia and 2D echocardiography was normal. Labs revealed anemia, thrombocytopenia, hypoglycaemia and hyponatremia. Workup for tropical infections, bacterial sepsis and other aetiologies of hypotension was negative. IV fluid resuscitation, empirical antibiotics, 25% dextrose infusion, 3% hypertonic saline and IV hydrocortisone were administered. The patient improved with these therapies and was referred to internal medicine department after 24 hours. Hormonal panel was suggestive of anterior pituitary hormonal deficiency (secondary hypothyroidism, secondary hypocortisolism, low serum prolactin and low gonadotropins). Pathogenic pathway of shock, hypoglycaemia and hyponatremia was thus related to hypocortisolism, anaemia and thrombocytopenia secondary to hypothyroidism, amenorrhoea due to low gonadotropins, and other non-specific features secondary to low prolactin levels and hypothyroidism. MRI brain showed an involuted pituitary gland with CSF filling the sella turcica (Figure 3). Subsequently, she was discharged on oral prednisolone, oral levothyroxine, anti-anxiety medications and high salt diet.

## Case 4: Postmenopausal female with recurrent hyponatremia

A 54-year-old post-menopausal female was admitted to ICU with altered sensorium and history of recurrent hyponatremia. Her last delivery was complicated with PPH. She had regular periods up to 47 years, however she experienced lactation failure after 3 months postpartum. First episode of hyponatremia was 15 years ago associated with a diarrhoea episode. Lowest serum sodium reported was 100 mEq/ L, with symptoms like fatigue and anorexia, which progressed to altered sensorium requiring ICU admission. Second episode of hyponatremia happened 3 years ago, when she had respiratory infection. Lowest sodium reported then was 126 mEq/ L which again imposed ICU admission. Third time was the current admission when she had serum sodium of 127 mEq/ L with altered sensorium, hypotension and bradycardia. Clinical improvement was noted after IV fluids, 3% hypertonic saline infusion and steroids. Hormone profile revealed anterior pituitary hormone deficiency (hypocortisolism and hypothyroidism) which explains the clinical improvement after stress doses of IV hydrocortisone, intravenous isotonic saline and oral levothyroxine. Clinical symptoms warranting ICU admission were related to hypocortisolism and hypothyroidism that improved after stress doses of IV hydrocortisone, intravenous isotonic saline and oral levothyroxine. MRI brain (image not shown) showed hypoplasia of pituitary with CSF filling the sella turcica, suggestive of empty sella. Later, she was continued on replacement doses of oral steroids and oral levothyroxine.

## Case 5: Postmenopausal female with severe hyponatremia and seizures

A 53-year-old postmenopausal female presented to ED with 3 episodes of generalized tonic clonic seizures (GTCS) for the past one day. She had no history of other comorbidities except for hypertension that was treated with cilnidipine 10 mg per day. Past history revealed amenorrhoea since her last delivery 18 years ago, which was complicated by PPH. Lab tests revealed hyponatremia (118 mEq/L) and hence an endocrine work up was initiated. EEG was normal. Hormonal investigation was suggestive of anterior pituitary hormone deficiency. She was initially started on anti-epileptics, stress doses of steroids and levothyroxine. Hyponatremia and altered sensorium were explained by hypothyroidism and hypocortisoloism. Anti-epileptics were gradually tapered off after a few months as there was no recurrence of seizures. Ultrasound abdomen revealed mild left sided pleural effusion likely due to hypothyroidism, which subsided spontaneously. MRI brain showed an empty sella (image not shown). She was subsequently discharged on replacement doses of oral prednisolone and oral levothyroxine.

**Table 1** describes the case descriptions (clinical presentation and lab values). **Table 2** shows the hormonal profile of these patients.

**Table 1:** Case descriptions.

	Case 1	Case 2	Case 3	Case 4	Case 5
Demographics					
Age (years)	41	34	28	54	53
Weight (Kg)	65	45	56	47	53
Height (cm)	150	148	147	159	144
Co-morbidities	Epilepsy	No	No	No	Systemic hypertension
PPH	Yes	Yes	Yes	Yes	Yes
Hysterectomy	Yes	Yes	No	No	No
Amenorhoea	Yes	Yes	Yes	No	Yes
Agalactia	Yes	Yes	Yes	Yes	No
Delay in diagnosis post-PPH (years)	8	3	2	20	18

Clinical presentation					
Encephalopathy	Yes	Yes	Yes	Yes	Yes
Hypotension	Yes	Yes	Yes	Yes	No
Vasopressors	Yes	Yes	No	No	No
Bradycardia	Yes	Yes	Yes	Yes	No
LV dysfunction (2D ECHO)	Yes (LVEF 40%)	Yes (LVEF 30%)	No	No	No
MV	No	Yes	No	No	No
AKI	Yes	No	No	No	No
Polyuria	Yes	No	No	No	No
Hypoglycemia	Yes	No	Yes	Yes	No
Hematological dysfunction	Yes	No	Yes	Yes	No
Labs at admission					
Hb (g/dl)	7.4	10.4	9.3	8.8	12.3
TLC ( /mm3)	3800	5700	6100	11000	4500
Platelets (lacs/ mm3)	0.75	5.1	0.75	5.5	3.5
Urea/Creatinine (mg/dl)	21/1.0	22/0.5	-/0.7	28/0.6	-/0.5
Na (mEq/L)	119	131	113	127	118
K (mEq/L)	4.2	4.04	3.84	5.0	3.3
T Bil	0.61	0.35	-	0.68	0.83
D bil	0.31	0.25	-	0.30	0.39
SGOT/SGPT	93/47	24/10	/90	24/29	104/34
ALP	411	219	-	112	35
Procalcitonin (micg/L) (N: 0-0.05)	0.11	0.73	-	-	-
Pro-BNP (pg/ml) (N: 0-125)	294	7880	-	-	-
USG abdomen	Post-hysterectomy with bilateral adnexal mass with complex septated cystic lesion.	Post-hysterectomy with very minimal pockets of collection. Abdominal drain in position.	Small uterus size	-	Mild left sided pleural effusion

PPH=Postpartum haemorrhage, MV=Mechanical ventilation, LV dysfunction=Left ventricular dysfunction, LVEF=Left Ventricular Ejection fraction, AKI=Acute Kidney Injury, Polyuria=urine output > 3 Litres/ day, Hypoglycaemia=RBS < 70 mg/dl, 2D ECHO=2 dimensional echocardiography, Hb= Haemoglobin, TLC=Total Leukocyte count, Na=Serum sodium, K=serum potassium, T Bil=Total Bilirubin, D Bil=Direct bilirubin, SGOT/SGPT=Serum glutamate oxaloacetate transaminase/Serum glutamate pyruvate transaminase, ALP=alkaline phosphatase, PRO-BNP=PRO-Brain Natriuretic Peptide, N=normal, - data not available

**Table 2:** Hormonal profile at admission.

Hormones	Baseline values	Case 1	Case 2	Case 3	Case 4	Case 5
Human GH by CLIA (ng/ml)	Females: 0-8	-	-	**<0.05**	**-**	**-**
LH (mIU/ml) by CMIA	Normally menstruating females:	4.9	**0.01**	**0.29**	**-**	**-**
	Follicular phase: 2.39-6.60					
	Mid-cycle peak: 3.06-74.24					
	Luteal phase: 0.9-9.33					
	Post-menopausal females: 10.39-					
	64.57					

FSH (mIU/ml) by CMIA	Normally menstruating females:	9.92	**0.06**	**1.25**	**2.2**	**1.01**
	Follicular phase: 3.03-8.08					
	Mid-cycle peak: 2.55-16.69					
	Luteal phase: 1.38-5.47					
	Post-menopausal females: 26.72-					
	133.41					

Estradiol (pg/ml) by CLIA	Normally menstruating females:	**-**	**-**	**< 10**	**-**	**-**
	Follicular phase: 21-251					
	Mid-cycle peak: 38-649					
	Luteal phase: 21-312					
	Post-menopausal females:					
	Not on HRT: < 10-28					
	On HRT: < 10-144					

ACTH (pg/ml) by CLIA		**-**	**-**	**-**	**15**	**-**

Morning serum cortisol (micg/dl) by CMIA	5-23	**3.3**	**2.2**	**0.1**	**3.69 (15.9 stimulated)**	**0.83 (6.36 stimulated)**

Thyroid profile by CLIA					**-**	**-**
T3 (ng/ml)	0.58-1.59	**0.25**	**0.49**	**0.41**		
Free T4 (ng/dl)	0.8-1.8	**-**	**-**	**0.17**	**0.67**	**-**
T4 (micg/dl)	4.87-11.72					
TSH (microIU/ml)	0.35-4.94	**0.97**	**1.67**	**1.91**	**-**	**3.17**
		**1.11**	4.08	**0.22**	**1.99**	**3.6**

Prolactin (ng/ml) by CLIA	Females:	0.76	0.88	1.5	8.8	-
	Non-pregnant: 2.8-29.2					
	Pregnancy: 9.7-208.5					
	Post-menopausal: 1.8-20.3					

LH=Luteinizing Hormone, FSH=Follicle Stimulating Hormone, TSH=Thyroid Stimulating Hormone, ACTH=Adrenocorticotropic hormone, GH=Growth Hormone, HRT= Hormone Replacement Therapy, CLIA=Chemi Luminescent Immunoassay, CMIA=ChemiLuminescent Microparticle Immunoassay, - not done, bold hormonal values represent hormonal deficiencies

## Discussion

Sheehan syndrome is a life-threatening endocrine emergency, resulting from postpartum pituitary necrosis secondary to severe PPH. Increased pituitary size, small sella turcica, autoimmunity to pituitary antigens, hypercoagulation and thrombosis of pituitary arteries and shock and vasospasm of pituitary arteries are all implicated in pathogenesis of Sheehan’s syndrome [8].

The clinical presentation may be either chronic or acute. The characteristic presenting features are agalactia and failure to resume menstruation in postpartum period. Usually, the evolution of the disease is chronic with subtle and non-specific features [9] like fatigue, headache, nausea, vomiting, psychiatric features [10] and depressive disorders. But, as the disorder progress, patients may present in ER with severe organ dysfunctions like coma, hypotension, dyselectrolytemias (eg hyponatremia), hypoglycaemia, seizures, pancytopenia, rhabdomyolysis, coagulopathy and renal failure [6]. In view of varied spectrum of presentation, a meticulous approach is warranted for a timely diagnosis.

The patients resemble partial or total hypopituitarism. Anterior pituitary hormones are affected more as compared to posterior pituitary hormones because posterior pituitary hormones are released directly from the hypothalamus to pituitary gland through the neurohypophysis. In hormonal tests, either the baseline hormonal values are low or dynamic assessment of these hormones reveal decreasing trends depending upon progression of disease [5,7]. Inapparent hormonal deficiency can also be unmasked by stimulation tests. There is no fixed pattern of hormonal deficiency, but lactotrophs and somatotrophs are the most common deficiencies as compared to other hormones due to their anatomical location in the pituitary gland.

In radiological evaluation by MRI brain, changes in pituitary gland have been found to vary consistent with the disease progression. In early stages, pituitary gland may be normal or even enlarged as a consequence of pregnancy-induced changes; later, it may shrink and get replaced completely by a cyst like structure. However, no correlation has been seen between MRI brain findings and the degree of hypopituitarism.

Essential criteria for diagnosis Sheehan’s syndrome include history of severe PPH, especially connected with delivery, at least 1 pituitary hormone deficiency and partial or total empty sella on MRI brain scan in chronic phase [11].

In previous case series,[11, 12, 13, 14] following clinical, hormonal and radiological findings have been noted; Age of patients: 28-70 years, PPH: 99-100%, delay in diagnosis from PPH: around 1 -33 years, non-specific symptoms: 52%, agalactia: 74-94%, amenorrhoea: 33-82%, partial hypopituitarism: 45%, panhypopituitarism: 55%, lactotroph (Prolactin) and gonadotroph [Luteinizing hormone (LH), follicle stimulating hormone (FSH)] failure: 80-100%, corticotroph [Adrenocorticotrophic hormone (ACTH)] failure: 16-76%, thyrotroph [Thyroid stimulating hormone (TSH)] failure: 16-83%. At times, TSH may be normal or even mildly elevated, which is inappropriate for the very low levels of free T4 [15]. In MRI brain, following findings have been noted; empty sella: 28-74%, partially empty sella: 33%, slight pituitary involution: 7% and normal pituitary: 18%. If we review the individual case reports on Sheehan’s syndrome, similar pattern of clinical, hormonal and radiological picture is seen as in our cases [3,16, 17, 18, 19, 20, 21]. **Table 3** shows the individual case reports on Sheehan’s syndrome.

**Table 3:** Case reports of Sheehan’s syndrome (SS) from India and other countries.

Age/PPH/Delay			Clinical presentation					Hormonal profile									Radiology	
Year; Country; Age of patient	PPH	Delay (yrs)	Agalactia	Menstrual problem	Non-specific	Organ failures	Na	GH	PRL	Hypothyroidism			Hypogonadism		Adrenal insufficiency		Pituitary size	Sella volume (ml)
										TSH	fT3	fT4	LH	FSH	ACTH	Cortisol		
2020;^16^ India 35y/F	yes	4	yes	yes	yes	Asthenia, anaemia	↓	-	↓	↓	↓	↓	↓/N	↓	-	↓	↓	-

2019;^17^ India 36y/F	yes	7	yes	yes	yes	Myxoedema coma, Coagulopathy, Pancytopenia, Hypoglycaemia	↓	-	-	N	↓	↓	↓/N	↓/N	-	↓	**-**	Partially empty sella

2016; ^18^ India 32y/F	yes	1	yes	yes	yes	Anaemia,	-	-	-	↓	-	-	-	↓	-	↓	**-**	Empty sella

2013; ^19^ India 41y/F	yes	22	yes	yes	yes	Hypoglycaemia Asthenia	↓	-	-	-	↓	↓	-	-	↓	↓	**-**	-

2019;^3^ USA 36y/F	yes	8	yes	yes	yes	Chronic pain upper and lower limbs, Hypoglycaemia	↓		↓	N	↓	↓	↓	↓	-	↓	CSF in pituitary fossa	Empty sella

2013;^21^ Italy 64y/F	-	30	-	-	yes	Rhabdomyolysis, hypothyroidism	**-**		-	-	-	-	-	-	-	↓	Atrophy	-

2013;^22^ Nepal 38y/F	yes	5	-	yes	yes	Sparse hair, Dyspnoea, Bradycardia, Shock	↓		↓	↓	N	↓	↓	↓	-	↓	**-**	Empty sella

PPH=Postpartum haemorrhage, Delay = diagnostic delay from PPH to diagnosis of Sheehan’s syndrome, Na=Serum Sodium, GH=Growth hormone, PRL=Prolactin, TSH=Thyroid stimulating hormone, fT3=free Triiodothyronine, T4=levothyroxine, FT4=free levothyroxine LH=Leutenizing hormone, FSH=Follicle stimulating hormone, ACTH=Adrenocorticotrophic hormone, MRI=Magnetic resonance imaging (MRI) brain, N=Normal, =Postpartum haemorrhage, Delay = diagnostic delay from event to presentation, Na=Serum Sodium, GH=Growth hormone, PRL=Prolactin, TSH=Thyroid stimulating hormone, fT3=free Triiodothyronine, T4=levothyroxine, FT4=free levothyroxine LH=Leutenizing hormone, FSH=Follicle stimulating hormone, ACTH=Adrenocorticotrophic hormone, MRI=Magnetic resonance imaging brain, N=Normal, ↓= decrease, mnths=months, d=day,- = data not available

In our patients, clues for diagnosis of Sheehan’s syndrome were history of PPH, agalactia and secondary amenorrhoea in almost all cases. Most of the patients had hemodynamic instability (hypotension, bradycardia), hyponatremia, hypoglycaemia and neurological dysfunction (seizures, altered sensorium) at ICU admission. In hormonal assessment, thyrotroph and corticotroph failure were seen in all the cases followed by lactotroph and gonadotroph failure in 4/5 (80%) cases. Growth hormone level could be evaluated in one patient only (case 3) and was found to be low. Other workups for tropical infections, sepsis, hyponatremia, hypoglycaemia etc. were negative. Etiologically, the clinical presentation of shock, hypoglycaemia and hyponatremia could be attributed to hypocortisolism, amenorrhoea to low gonadotropins, agalactia to low prolactin levels and other non-specific symptoms (headache, fatigue, anorexia) to growth hormone deficiency and - INCONCLUSIVE - they might be intricated consequences of hypocortisolism and hypothyroidism. Radiologically, an empty sella turcica with involuted pituitary gland in MRI brain was seen in all the cases. Thus, clinical, hormonal and radiological findings led to diagnosis of Sheehan’s Syndrome [11]. Diagnostic delay from symptom onset ranged from 2-20 years. All patients improved on hormone replacement therapies.

## Conclusion

Sheehan’s syndrome is an important and often under recognised cause of hypopituitarism in developing countries, which may present as an endocrine emergency in postpartum females secondary to ischemic postpartum pituitary necrosis. The criteria for diagnosis may include agalactia, amenorrhoea, hypotension, bradycardia, hypoglycaemia and hyponatremia in patients with a background history of PPH. A meticulous obstetric history, clinical evaluation along with hormonal and radiological assessment are essential to avoid misdiagnosis. Timely diagnosis, appropriate supportive management for organ failures in ICU or emergency settings and long term hormonal replacement therapy are essential to improve the outcome of patients.
